# Revisiting the Geographical Distribution of Thyroid Cancer Incidence in Fukushima Prefecture: Analysis of Data From the Second- and Third-round Thyroid Ultrasound Examination

**DOI:** 10.2188/jea.JE20210165

**Published:** 2022-12-05

**Authors:** Tomoki Nakaya, Kunihiko Takahashi, Hideto Takahashi, Seiji Yasumura, Tetsuya Ohira, Hiroki Shimura, Satoru Suzuki, Satoshi Suzuki, Manabu Iwadate, Susumu Yokoya, Hitoshi Ohto, Kenji Kamiya

**Affiliations:** 1 Graduate School of Environmental Studies, Tohoku University, Miyagi, Japan; 2 Radiation Medical Science Center for the Fukushima Health Management Survey, Fukushima Medical University, Fukushima, Japan; 3 Department of Biostatistics, M&D Data Science Center, Tokyo Medical and Dental University, Tokyo, Japan; 4 National Institute of Public Health, Saitama, Japan; 5 Department of Public Health, Fukushima Medical University School of Medicine, Fukushima, Japan; 6 Department of Epidemiology, Fukushima Medical University School of Medicine, Fukushima, Japan; 7 Department of Laboratory Medicine, Fukushima Medical University School of Medicine, Fukushima, Japan; 8 Department of Thyroid and Endocrinology, Fukushima Medical University School of Medicine, Fukushima, Japan; 9 Thyroid and Endocrine Center, Fukushima Medical University, Fukushima, Japan; 10 Research Institute for Radiation Biology and Medicine, Hiroshima University, Hiroshima, Japan

**Keywords:** thyroid cancer screening, discrete survival analysis, flexibly shaped spatial scan statistics, maximized excess events test (MEET)

## Abstract

**Background:**

After the first-round (Preliminary Baseline Survey) ultrasound-based examination for thyroid cancer in response to the accident at the Fukushima Daiichi Nuclear Power Plant in 2011, two rounds of surveys (Full-scale Survey) have been carried out in Fukushima Prefecture. Using the data from these surveys, the geographical distribution of thyroid cancer incidence over 6 or 7 years after the disaster was examined.

**Methods:**

Children and adolescents who underwent the ultrasound-based examinations in the second- and/or third-round (Full-scale) survey in addition to the first-round survey were included. With a discrete survival model, we computed age, sex, and body mass index standardized incidence ratios (SIRs) for municipalities. Then, we employed spatial statistics to assess geographic clustering tendency in SIRs and Poisson regression to assess the association of SIRs with the municipal average absorbed dose to the thyroid gland at the 59-municipality level.

**Results:**

Throughout the second- and third-round surveys, 99 thyroid cancer cases were diagnosed in the study population of 252,502 individuals. Both flexibly shaped spatial scan statistics and maximized excess events test did not detect statistically significant spatial clustering (*P* = 0.17 and 0.54, respectively). Poisson regression showed no significant dose-response relationship: the estimated relative risks of lowest, middle-low, middle-high, and highest areas were 1.16 (95% confidence interval [CI], 0.52–2.59), 0.55 (95% CI, 0.31–0.97), 1.05 (95% CI, 0.79–1.40), and 1.24 (95% CI, 0.89–1.74).

**Conclusion:**

There was no statistical support for geographic clustering or regional association with radiation dose measures of the thyroid cancer incidence in the cohort followed up to the third-round survey (fiscal years 2016–2017) in Fukushima Prefecture.

## INTRODUCTION

The accident at the Fukushima Daiichi Nuclear Power Plant (FDNPP), caused by the Great East Japan Earthquake of March 11^th^, 2011, resulted in the release of radioactive material into the atmosphere and raised concerns about the risk of thyroid cancer in children and adolescents exposed to radioactive iodine, similar to the accident at Chernobyl Nuclear Power Plant (CNPP) in 1986. In response to this situation, the Fukushima Health Management Survey (FHMS), which included an ultrasound-based screening program for thyroid cancer (thyroid ultrasound examination [TUE]), was launched in order to monitor the long-term health of the children and adolescents in Fukushima Prefecture.^1^^,^^2^

The first-round (Preliminary Baseline) survey, scheduled to run for 3 fiscal years from October 2011, was conducted to establish the baseline information of thyroid cancer prevalence for subsequent surveys. Since the exposure dose of radioactive iodine in Fukushima was estimated as far smaller than that from the CNPP accident, it was expected that an increased occurrence of thyroid cancer was unlikely.^3^^,^^4^ Nevertheless, the first-round survey diagnosed 116 thyroid cancers (including suspected cases), once again raising the question of a link between radiation and thyroid cancer^5^ as well as the possibility of overdiagnosis without clinical symptoms by the introduction of ultrasonography to a large population survey.^6^ As summarized by the recently updated review by United Nations Scientific Committee on the Effects of Atomic Radiation (UNSCEAR),^7^ most studies examining the geographical distribution of thyroid cancer detection rates (understood as the prevalence rate) in the first-round survey did not show dose-response relationships with regional differences in radiation dose or any statistical spatial clustering of thyroid cancer detection rates.^8^^–^^10^

TUEs were continued after the first-round (Preliminary Baseline) survey at 2-year intervals from 2014 onwards. To date, the results of the second- (fiscal years 2014–2015) and third- (fiscal years 2016–2017) rounds of the survey (Full-scale Survey) have been finalised.^11^ Using the results of the second-round survey, two studies reported no statistically significant association between the estimated radiation dose and thyroid cancer diagnosis.^12^^,^^13^ Some studies also indicated that the detection rates of thyroid cancer in the second-round survey were associated with the mean external radiation dose in air based on published regional counts of thyroid cancer cases,^14^^,^^15^ although the results could not be adjusted for the age at diagnosis and prevalence and incidence could not be distinguished.^16^

Importantly, assessments of thyroid cancer incidence should be evaluated through multiple surveys based on the observation interval period between them. In the European areas affected by CNPP accident, the number of thyroid cancer cases increased rapidly from 4 years after the accident, and the incidence of thyroid cancer among children (under 15 years of age) peaked at 10 years after the accident,^17^ but studies using the second-round survey evaluated the situation only 3 or 4 years after the FDNPP accident.

This study thus aimed to statistically examine the geographical variability in thyroid cancer incidence among children and adolescents in Fukushima Prefecture over longer periods compared to previous studies by assessing the data up to the third-round TUE (ie, 6 or 7 years after the accident). Spatial cluster detection methods and Poisson regression combined with a discrete survival model were employed to identify areas with excess risk of thyroid cancer incidence and the potential association of geographic indicators of radiation doses with the distribution of thyroid cancer incidence after the first-round TUE.

## METHODS

### Study participants

The Fukushima Medical University conducted the TUEs and compiled the database of the examination results. The dataset as of December 8^th^, 2020 was the data source for this study. The target population for the first-round TUE included children and adolescents (<19 years of age at the time of the accident), which was then expanded in the second-round TUE to include children who were born from April 2, 2011 to April 1, 2012. In the third-round TUE, the current scheduling plan involves testing persons up to the age of 20 years once every 2 years and testing once every 5 years for those who are older. The sizes of the registered population in the first-, second-, and third-round TUEs were 367,579, 359,184, and 345,544, respectively. In this study, we focused on persons who lived in Fukushima Prefecture when the FDNPP accident occurred and who had been tested in the first-round TUE and no thyroid cancer were diagnosed, and at least one of the two subsequent TUEs, resulting in 359,206 target subjects.

Each TUE involved two stages. The primary examination involved ultrasonography; if large nodules (size, 5.1 mm or more) or cysts (size, 20.1 mm or more) were found, or if a definitive diagnosis was clinically required, confirmatory examination by fine-needle aspiration cytology (FNAC) was recommended to confirm the malignancy. We regarded the malignant or highly suspicious of malignancy diagnoses based on confirmatory testing as a diagnosis of thyroid cancer for this study. The details of the TUE protocol are provided elsewhere.^18^ Written informed consent for the study was obtained from the parents of every participant. This study using the TUE database was approved by the Ethics Committee of the Fukushima Medical University (approval no. 1318).

### Statistical analysis

For this study, the standardized incidence ratio (SIR) at the municipality level for the study subjects (not diagnosed with thyroid cancer at the first-round TUE) was defined as:

$${sir}_{i}=o_{i}/e_{i},$$

where *i* is the index of the municipality based on the residential address at the time of the disaster. *o_i_* and *e_i_* are the number of observed and expected diagnosed cases at the second- and third-round TUEs, respectively. The expected number of cases was obtained from the estimated probability of thyroid cancer diagnoses using the following discrete survival model at the individual level:

$$e_{i}=\sum_{j\in S_{i}}\sum_{t=2}^{3}\hat{\lambda_{jt}}$$

$$y_{jt}∼\text{Bernoulli}(\lambda_{jt})$$

$$\log(-\log(1-\lambda_{jt}))=\log d_{jt}+\alpha +\sum_{k}\beta_{k}x_{jtk}$$

where *j* and *t* are the indexes of the subject and the round of TUE (either 2 or 3), respectively. *α* and *β_k_* are coefficients to be estimated. Suppose a pseudo-observation unit of the combination of the subject and the round of TUE. The unit is defined only for the case that the subject *j* who takes the *t*th TUE but is not diagnosed with thyroid cancer in the earlier TUE (s). *y_jt_* is defined as the diagnosis indicator that takes the value one if subject *j* was diagnosed at the *t*th TUE and zero otherwise. *d_jt_* is the interval length (in years) of examination dates between the examination date of the *t*th TUE and that of the most recent prior TUE. *x_jtk_* is the *k*th associated individual factor to be adjusted for the computation of SIR. Sex, age, body mass index (BMI)^19^ at the examination, and the dummy variables of the round of TUE were considered as the adjusting factors for this study. We regarded BMI <5 or BMI >50 as recording errors of the database and applied an available value from an other-round survey to the incorrect/missing values.

The exponential value of the coefficient, exp(*β_k_*), can be interpreted as the associated hazard ratio of one-unit increase in the explanatory variable as in the Cox proportional hazard model. The model can be fitted as a binomial generalized linear model with a complementary log-log link function by treating the *y_jt_* as independent Bernoulli observations with the probability of *λ_jt_* for subject *j* at the *t*th round TUE. This type of survival model is suitable for interval-based observations of event occurrence.^20^ The inclusion of the offset variable, log *d_jt_*, corresponds to the adjustment of person-years for a constant hazard model.

Since each pseudo-observation was modelled in the form of an independent Bernoulli trial with a rare probability of event occurrence, it should be reasonable to consider that the aggregated behaviors of the observation approximately follow a Poisson distribution, *o_i_* ∼ *Poisson*[*e_i_*]. To statistically test the geographical variation of the thyroid cancer incidence, we can postulate the following model:

$$o_{i}∼\text{Poisson}[r_{i}e_{i}],$$

where *r_i_* is the relative risk of thyroid cancer incidence in the *i*th municipality. The null hypothesis of our spatial analysis is that the relative risks are geographically homogeneous (*r_i_* = 1 for all *i*).

An alternative hypothesis based on the null hypothesis is that regions with a higher risk compared to the others existed. Tango and Takahashi^21^ proposed flexibly shaped spatial scan statistics (Flexscan) to search for irregularly shaped clusters of regions with elevated risk using likelihood ratio statistics to compare risks inside and outside possible topologically connected areas. The region with the highest likelihood ratio is called the ‘most likely cluster,’ whose statistical significance can be assessed using Monte Carlo simulation under the null assumption of homogeneous risk. Since the test statistic is obtained as the highest likelihood ratio in the entire study region, this method avoids the problem of multiple testing. Flexscan is expected to detect elevated risk regions along the areas with high doses and display them on the map, even if the true cluster is non-circular (eg, directionally biased distribution by the wind). For the technical details, see eMaterial 1.

A clustering tendency of incidence can also be identified as a positive spatial autocorrelation of the relative risks, meaning that SIRs are likely to be similar between areas with closer distance. Such clustering tendency may suggest that SIRs are spatially structured by the distribution of other geographic factors. Tango’s MEET^22^ has the highest power for detecting the general spatial clustering tendency of incidence rates.^23^ In this method, the clustering tendency is measured using the C-index with a pre-fixed scale parameter corresponding to the geographic size of clustering. Since testing the multiple C-index with different scale parameters in searching for the optimal (most probable) scale results in the multiple comparisons, MEET involves computing the multiple-comparison adjusted *P*-value of clustering with the aid of Monte Carlo simulation. For the technical details, see eMaterial 1.

**Figure 1.**
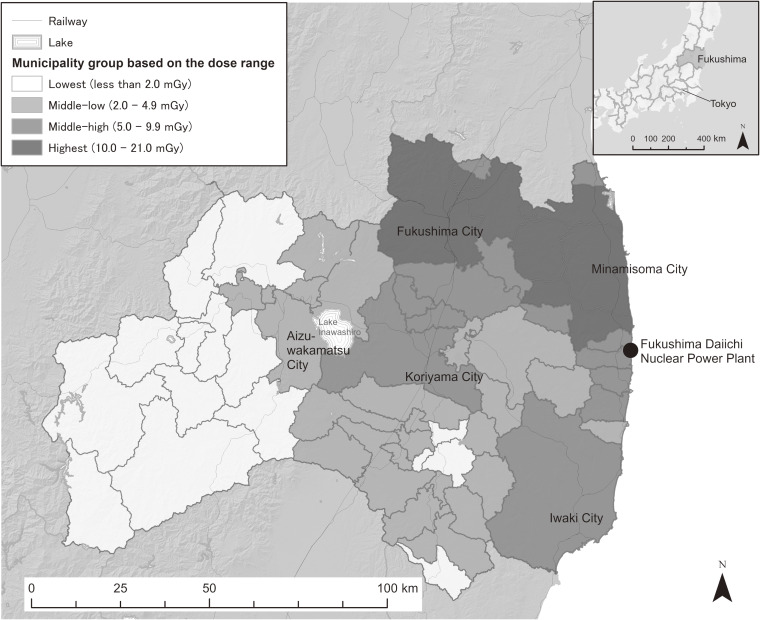
The four municipality-groups of the average absorbed dose to thyroid grand in the first year after the accident in Fukushima Prefecture, Japan, based on the UNSCEAR 2020 report.^7^

Regression modelling of geographic variables can be used to assess whether the relative risks are geographically structured with the other variable(s). Poisson regression shares the same assumption of the Poisson process for spatial clustering tests:

$$o_{i}∼\text{Poisson}[r_{i}e_{i}],$$

$$\ln(r_{i})=\sum_{k=1}^{4}\beta_{k}z_{k,i},$$

where *z**_k_*_,_*_i_* is the dummy variable of the *k*th dose-range group in the *i*th municipality (if the area belongs to the *k*th group, it takes one and otherwise zero). It should be noted that $\exp({\hat{\beta }}_{k})$ is the estimate of relative risk for the *k*th group. In accordance with a previous study,^13^ we referred to the UNSCEAR’s updated information of municipal average absorbed doses in the thyroid gland in the first year after the accident by considering both internal and external exposures. Since the currently available data in the report were only those for infants, we prepared four area groups based on these data (lowest: <2.0 mGy, middle-low: 2.0–4.9 mGy, middle-high: 5.0–9.9 mGy, highest: ≥10.0 mGy) by assuming that the area division reflects general degrees of radiation exposure to the residents (Figure 1). For the evacuated areas, estimates were given for various evacuation scenarios. The municipal representative number was obtained by determining the median of those scenarios for each municipality. For Minamisoma City, where residents of some parts were evacuated, the average dose number of the non-evacuated residents was treated as an estimate of a scenario and the median value for the municipality was obtained. Since the size of evacuees for each scenario was unavailable, each evacuation scenario was treated equally to calculate the median of absorbed radiation doses for each municipality.

All analyses were carried out in the R version 4.0.2 (R Foundation for Statistical Computing, Vienna, Austria) environment using the glm() function for the discrete survival and Poisson regression, the R package rflexscan version 0.4.0 for Flexscan, and Tango’s R script for MEET.^24^ In all analyses, the level of significance was set at *P* < 0.05.

## RESULTS

**Figure 2.**
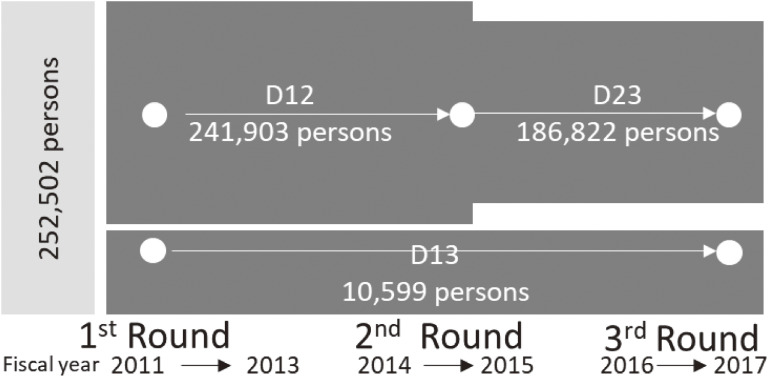
Diagram of the study population. Dxy, the subgroup that underwent thyroid screening examinations at the xth and yth round surveys; TUE, thyroid ultrasound examination.

Figure 2 presents a diagram of the study population subgroups in the dataset by different combinations of TUEs. The total population size was 252,502 (70.3% of the target population) after excluding 188 cases with incorrect/missing BMI values (incorrect/missing BMI values for 5,818 participants (2.3%) were replaced from values of an other-round survey). All study participants underwent the first-round TUE. Of this population, 241,903 participants received a second-round TUE (D12), and 186,822 received a third-round TUE (D23). A total of 10,599 participants did not receive the second-round TUE after the first but received the third (D13). Table 1 shows the summary of the study population composition. The D12, D23, and D13 subgroups included 69, 28, and 2 diagnosed cases of thyroid cancer. In comparison with the D12 participants who underwent the second-round TUE, the D23 and D13 participants who underwent the third-round TUE showed average inter-examination intervals of approximately 2 and 4 years, respectively, with a corresponding increase in the age distribution and a slight increase in the proportion of larger BMI values.

**Table 1.** Sample characteristics

		D12		D23		D13	
Sex	Men	121,551	(50.2%)	93,758	(50.2%)	5,356	(50.5%)
	Women	120,352	(49.8%)	93,064	(49.8%)	5,243	(49.5%)

Age, years	0–4	9,696	(4.0%)	0	(0.0%)	0	(0.0%)
	5–9	67,459	(27.9%)	42,945	(23.0%)	4,504	(42.5%)
	10–14	86,020	(35.6%)	75,780	(40.6%)	1,670	(15.8%)
	15–19	64,888	(26.8%)	59,075	(31.6%)	2,542	(24.0%)
	20–24	13,836	(5.7%)	8,224	(4.4%)	1,607	(15.2%)
	25–27	4	(0.0%)	798	(0.4%)	276	(2.6%)

BMI, kg/m^2^	5 to <15	31,189	(12.9%)	18,304	(9.8%)	1,659	(15.7%)
	15 to <20	134,620	(55.7%)	103,149	(55.2%)	5,442	(51.3%)
	20 to <25	63,950	(26.4%)	55,013	(29.4%)	2,815	(26.6%)
	25 to <30	9,635	(4.0%)	8,281	(4.4%)	500	(4.7%)
	30 to <50	2,509	(1.0%)	2,075	(1.1%)	183	(1.7%)

Interval, years	Minimum	0.019		0.071		1.159	
	1st Quantile	1.986		1.841		3.663	
	Median	2.088		2.005		4.049	
	Mean	2.126		2.031		4.025	
	3rd Quantile	2.252		2.197		4.397	
	Maximum	6.562		5.608		8.211	

Number of diagnosed thyroid cancer		69		28		2	
Total		241,903		186,822		10,599	

Dxy, the subgroup that underwent thyroid screening examination at the xth and yth round surveys.

**Figure 3.**
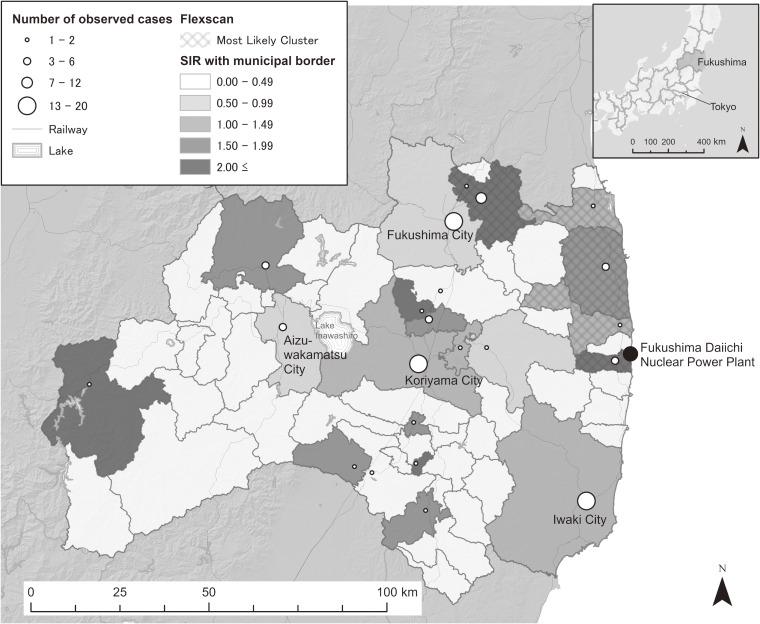
Standardized incidence ratio (SIR) and the most likely cluster of childhood and adolescent thyroid cancer cases derived from flexibly shaped spatial scan statistics in Fukushima Prefecture, Japan. The circles near the municipal town hall points show the municipal number of thyroid cancer cases. The most likely cluster, which is shown with the hatch diagonal stroke in the map, consists of six municipalities. Its relative risk and associated *P*-value are 2.00 (the observed and expected numbers of cases were 24 and 12.0, respectively) and 0.168.

**Figure 4.**
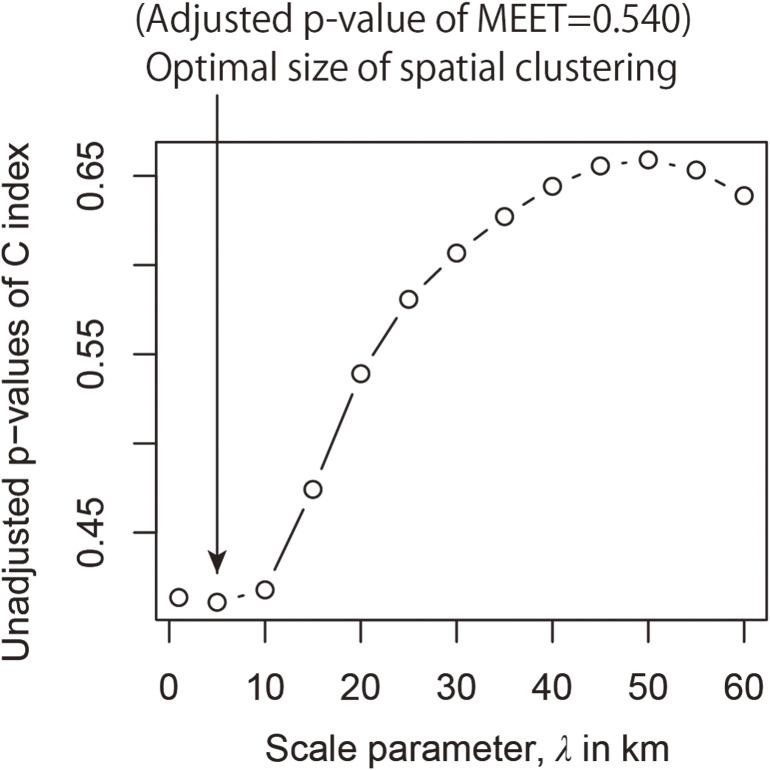
Scale profile of Tango’s spatial clustering test index (C-index) and adjusted *P*-value of the maximized excess events test (MEET)

Table 2 shows the results of the discrete survival model for the dataset. The negative estimate of the coefficient of the dummy variable for those undergoing the third-round TUE indicated that the hazard ratio of thyroid cancer was lower for the third-round TUE than that for the second-round TUE. As expected, age and BMI were positively associated with the hazard ratio of thyroid cancer detection. Based on the predicted values of the fitted model, we computed the municipality-level SIRs in the 59 municipalities in Fukushima Prefecture (Figure 3). The application of Flexscan to the dataset showed that the most likely clusters consisted of 6 municipalities around the north-eastern part of the prefecture to the north of the FDNPP, as shown in Figure 3. Although the relative risk of the cluster region was 2.0 (the observed and expected numbers of cases were 24 and 12.0, respectively), the *P*-value (0.167) associated with the cluster was not significantly low. MEET also did not detect a substantial clustering tendency in regional thyroid cancer incidence at all spatial scales tested. Figure 4 provides the profile of C-index at different spatial scales, indicating that the scale of a quite short distance, 5 km, attained the lowest *P*-value (the multiple-testing adjusted *P*-value of MEET: 0.540). This indicates that the municipal SIRs of thyroid cancer were not geographically clustered/structured.

**Table 2.** Results of the discrete survival model using complementary log-log binomial regression

	exp(Coef.)	95% CI (LL, UL)		*P*-value
*α* (intercept)	1.84 × 10^−12^	4.38 × 10^−12^	7.74 × 10^−9^	<0.001
*β* (round of TUE: second = 0, third = 1)	0.459	0.299	0.705	<0.001
*β* (Sex: men = 0, women = 1)	1.283	0.859	1.914	0.223
*β* (log(Age in years))	24.811	10.237	60.137	<0.001
*β* (log(BMI in kg/m^2^))	5.088	1.519	17.039	0.008

CI, confidence interval; Coef., estimate of coefficient; LL, lower limit; UL, upper limit.

*N* = 439,324, deviance = 1700.0.

The Poisson regression models did not detect statistically significant relationships between thyroid cancer incidence and regional mean estimated radiation dose (Table 3). According to the Akaike Information Criterion (AIC), the fitted model did not show any statistical improvement over the null model that assumed geographically homogeneous risks.

**Table 3.** Results of Poisson regression with estimated municipal groups based on the absorbed dose ranges

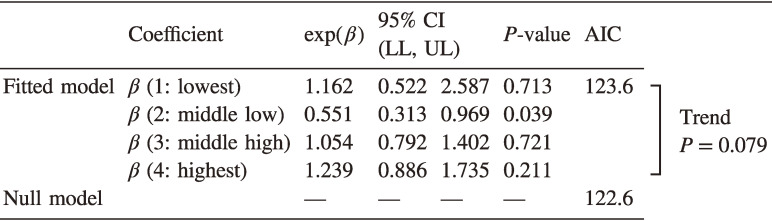

AIC, Akaike’s Information Criterion; CI, confidence interval; LL, lower limit; Null, the model without explanatory variables and coefficients; UL, upper limit.

*n* = 59.

## DISCUSSION

The results of the series of analyses in this study showed no substantial geographical variability in the incidence of thyroid cancer in Fukushima Prefecture after the first-round TUE. This is the first study to examine regional differences in the diagnosis rate of thyroid cancer using cohort samples who underwent the first-round TUE and following them until 6 or 7 years since the FDNPP accident in 2011. The long-term observation period increased the statistical power by increasing the number of diagnosed cases, the follow-up assessment of the first-round TUE recipients allowed analysis of incidence rather than prevalence, and individual-level adjustment for intervals between examinations (corresponding to person-years) and for sex, age, and BMI at the time of the examinations allowed us to assess regional differences in thyroid cancer diagnosis rates in a more reliable manner.

After the CNPP accident, there were several case-control studies of children and adolescents who had undergone ultrasound examinations in Belarus^25^ and Ukraine,^26^^,^^27^ suggesting strong radiation-related increased risks of thyroid cancer, such as 2.15 (95% CI, 0.81–5.47) excess odds ratio per Gy with the 0.56 Gy mean thyroid dose for the participants in the Belarus study.^25^ According to the updated UNSCEAR’s report,^7^ the absorbed doses to the thyroid gland of the whole population in Fukushima Prefecture were about two orders of magnitude smaller than those in Belarus after the CNPP accident. The mean estimated absorbed thyroid dose in the first year among the non-evacuee population in Fukushima Prefecture was about 5 mGy, and only 0.5% of them were estimated to have received >20 mGy. The low levels of radiation exposure may explain our statistically insignificant results.

Previous studies using the results of the second-round TUE to analyze the association between exposure dose estimates and the detection rates of thyroid cancer showed no significant association.^12^^,^^13^ We confirmed these findings using the third-round TUE data with a different approach. The AIC values suggested that the fitted Poisson models were statistically inferior to the null model, which assumed homogenous incidence over the region. It is possible that our adjustment for individual BMI attenuated the association between detection rate and radiation dose measures. Our analysis confirmed that a high BMI is associated with an increased hazard ratio for the development of thyroid cancer. However, the addition of the collective dose measurement variable at the individual level did not substantially change the coefficient for BMI.

Despite these statistically insignificant results, the relative risk estimates for different dose area-groups showed a marginal positive trend (*P* = 0.08), and the highest dose group had the highest relative risk (1.24; 95% CI, 0.89–1.74, *P* = 0.72). The most likely cluster of Flexscan overlapped with high radiation dose areas, including the municipalities in the vicinity of FDNPP. To interpret these results, care is needed in understanding the sensitivity of thyroid cancer detection without symptoms in the ultrasound examination. Although the sensitivity of diagnosis by testing was assumed to be the same everywhere for the statistical analysis, the implementation of FNAC, which confirms the diagnosis of thyroid cancer, involved subjective judgments by doctors and patients, suggesting that the procedure was most likely performed at times and in areas where the risk was perceived to be high.^13^^,^^16^ This may result in higher detection rates in some areas with higher absorbed doses.

Moreover, the short half-life of radioactive iodine-131 (approximately 8 days) made it difficult to estimate the accurate geographic distribution of radiation exposures. Toki et al^14^ reported that iodine-131 in soil was not correlated with the detection rate of thyroid cancer at the second-round survey, but soil-based measurements were not fully available for all municipalities in the prefecture. In this study, we tested for the existence of general geographical clustering of incidence rates by Flexscan and MEET, without identifying the regions with potentially high incidence rates in advance, but none of the results was significant in terms of the heterogeneity of thyroid cancer incidence among the study municipalities. Considering the uncertainty of radiation dose for evacuation zones, we conducted a sensitivity analysis of Poisson regression by using minimum or maximum estimates among different scenarios for evacuated municipalities as the municipal representative radiation dose rather than the median values, resulting in similar results (eMaterial 2, eTable 1 and eTable 2). Additionally, we did not consider other sources of radiation exposure, particularly from computer tomography (CT) examinations whose exposure could be larger than those from the FDNPP accident. However, the decision to perform a CT examination would not be correlated to the municipality-level of radiation dose but could be considered to occur randomly, causing little bias.

In the CNPP accident, the incidence of thyroid cancer among children in the exposed areas remained high for approximately 30 years after the accident.^27^ Our results for the discrete survival model showed that the incidence of thyroid cancer decreased in the third-round TUE compared to the second-round TUE, even though it was still approximately 7 years after the FDNPP accident. This finding indicated that there was no increasing trend of thyroid cancer in Fukushima. One possibility could be that the third-round TUE could have been more restrained in the implementation of confirmatory testing, because of the potential overdiagnosis^6^ from the results of the first-round TUE.

In another study using a mathematical model of thyroid cancer development,^28^ the sensitivity of ultrasound screening at the first-round TUE was estimated to be around 0.6–0.7, suggesting that some of the prevalent cases at the first-round TUE may not have reached the indication of FNAC and detected at the second- or third-round TUEs, which may also explain the reduced incidence in the third-round TUE under the assumption that there was essentially no radiation-induced thyroid cancer incidence.

This study had several limitations. First, in this study, the tests of spatial clustering and Poisson regression were based on the results of estimating the expected number of municipalities in advance by the discrete survival model, so the uncertainty inherent in the estimation of the expected number was not taken into consideration for the spatial analysis. As a result, the *P*-value of the tests may have been underestimated, but no significant clustering tendency was found. While this study used Poisson regression to analyze the geographic relationship between the absorbed dose and incidence in the style of an ecological analysis, it could also be conducted as an individual-level analysis by adding the municipality-level absorbed-dose group variables to the individual-level discrete survival analysis used to estimate the expected values, resulting in a virtually identical result (see eMaterial 3, eTable 3, and eTable 4).

Second, as argued above, there would remain potential biases in the observed incidence. It is necessary to conduct a more detailed analysis of the temporal and geographical differences in the confirmatory testing implementation of FNAC to evaluate the detection rates of thyroid cancer.^29^ It is also important to note that the number of people who have not been examined by TUE is on the increase, and that follow-up assessments for individuals aged ≥18, who often move out of the region when they graduate from high school, are particularly getting more difficult to follow in later TUEs. Although we adjusted for age in our analysis, we cannot deny the possibility that there is still a bias due to the number of people who have not been examined. Furthermore, our analysis may depend on the size and shape of municipalities used to estimate the level of absorbed radiation doses. Spatially aggregated measurements reducing the exposure variability may result in larger uncertainty in estimating the radiation effects.^30^ Hence, it is desirable to verify our study result using individual-level measurements analysis of both internal and external radiation doses.

Third, considering the primary purpose of the FHMS thyroid screening as a health-supportive program, it would be desirable to account for wider contextual factors, such as phycological stress,^14^ rather than only radiation effects. An increase in BMI owing to a reduction in outdoor exercise due to radiation anxiety^31^ may increase the risk of thyroid cancer in the longer term. In this case, instead of using BMI as an adjustment factor, it is important to carefully monitor regional differences in changes in BMI among the children and adolescents in Fukushima Prefecture.

In conclusion, our analysis of cohort data combining information from three thyroid examinations from fiscal years 2011 to 2017 showed no substantial geographic variation in the observed incidence of thyroid cancer after the FDNPP accident among municipalities in Fukushima Prefecture, and no statistical support for a positive dose-response relationship to the updated regional estimates of absorbed dose.

## SUPPLEMENTARY MATERIAL

eMaterial 1. Technical details

eMaterial 2. Sensitivity analysis of Poisson regression with minimum and maximum radiation-dose estimates for evacuated municipalities

eTable 1. Results of Poisson regression with estimated municipal groups based on the absorbed dose ranges with minimum estimates among different scenarios for evacuation zones

eTable 2. Results of Poisson regression with estimated municipal groups based on the absorbed dose ranges with maximum estimates among different scenarios for evacuation zones

eMaterial 3. Comparison of rate ratio estimations of incidence between Poisson regression and the complementary log-log binomial model

eTable 3. Result of the Poisson regression with estimated municipal groups based on absorbed dose ranges (estimates of rate ratios)

eTable 4. Results of the discrete survival model using complementary log-log binomial regression with the variable of estimated municipal groups based on absorbed dose ranges
