# Supplementary material for: Revisiting the Geographical Distribution of Thyroid Cancer Incidence in Fukushima Prefecture: Analysis of Data From the Second- and Third-round Thyroid Ultrasound Examination
Source: J Epidemiol. 2022 Dec 5;32(Suppl 12):S76–83. doi: 10.2188/jea.JE20210165 (PMC9703926; doi:10.2188/jea.JE20210165)
Supplement: Supplementary file 1 [file je-32-S076-s001.pdf]

eMaterial 1. Technical details

Flexibly shaped spatial scan statistics (Flexscan)

Suppose that o_i and e_i are the observed and expected number of cases in region i, and the observed cases were generated from a Poisson distribution. Under the condition that there are only two different risks inside and outside the window region, w, the likelihood ratio function, L_w, to evaluate if the two risks are equivalent can be defined as follows:

$$L_w = \left(\frac{o_i}{e_i}\right)^{o_i} \left(\frac{N - o_i}{N - e_i}\right)^{(N-o_i)} I(o_i > e_i)$$

where $N(= \sum_i o_i = \sum_i e_i)$ is the total number of cases, and I is the indicator function which is one in case of $o_i > e_i$ or zero otherwise. The test statistic, S, is defined as the maximum of the likelihood ratio among the possible set of window regions.

$$S = \max_w L_w$$

If the value of S is higher than the critical value of the preset alpha level obtained from the Monte Carlo simulation under the null distribution, the most likely cluster is considered to be significant at the alpha level.

Flexscan considers topologically connected regions a window, and searches exhaustively for all the possible combinations of regions for each focusing unit, with the maximum length of topological connection.[S1] The size of the maximum connection is set at 15. The number of Monte-Carlo simulations run was set at 9,999.

Maximized Excess Events Test (MEET)

An index of spatial clustering, C, depending on the scale parameter λ is defined as follows:

$$C(\lambda) = \sum_{i,j} (o_i - e_i)(o_j - e_j) \exp\left(-4\frac{d_{ij}^2}{\lambda^2}\right)$$

where d_{ij} is the distance between the municipalities i and j. In this study, the distance is calculated by the Euclidean distance between town hall locations on March 11, 2011. The scale parameter λ controls the cluster size to be detected in a geographic space. A smaller value is sensitive to small spatial clustering while a larger value is more sensitive to larger clustering. MEET was proposed to

provide the adjusted P-value of the statistics to take into account multiple testing for the scale parameter as follows:

$$P_{min} = \min_{0<\lambda\leq\lambda_{max}} Pr\{C(\lambda) > c(\lambda)|H_0, \lambda\}$$

where c is the observed value of C. For practical implementation, 'line search' for the optimal lambda is recommended for predefined series of λ, and the probability of testing is obtained by Monte Carlo simulation under the null distribution. Tango suggested max $d_{ij}/4$ as the upper limit of line search.[S2] In this study, we considered 13 values of $\lambda = \{0.1, 5, 10, 15, \ldots, 50, 55, 60\}$. The number of Monte-Carlo simulation runs was set as 9,999.

SUPPLEMENTAL REFERENCES

S1. Tango T, Takahashi K. A flexibly shaped spatial scan statistic for detecting clusters. International Journal of Health Geographics. 2005;4:11.

S2. Tango T. A test for spatial disease clustering adjusted for multiple testing. Statistics in medicine. 2000;19:191-204.

eMaterial 2. Sensitivity analysis of Poisson regression with minimum and maximum radiation-dose estimates for evacuated municipalities

Poisson regression models were fitted for assessing relationships between municipal thyroid cancer incidence and regional absorbed radiation dose. For the evacuated areas, there was significant variation in the radiation dose estimates depending on how the residents were evacuated. Table 3 in the main body of this article shows the result of a Poisson regression using the median value of the estimates by evacuation scenario presented in the UNSCEAR 2020 report as the regional absorbed radiation dose for the municipalities included in the evacuated areas. The supplementary tables, eTable 1 and eTable 2, show the results of Poisson regression using the minimum and maximum value of the estimates by evacuation scenario as the municipal absorbed radiation dose for the evacuated areas, respectively. In this sensitivity test, neither of the fitted models using the minimum and maximum values showed a statistically significant relationship between thyroid cancer incidence and absorbed radiation dose. In addition, both fitted models did not achieve any statistical improvement in terms of AIC over the null model, which assumed a geographically homogeneous risk.

eTable 1. Results of Poisson regression with estimated municipal groups based on the absorbed dose ranges with minimum estimates among different scenarios for evacuation zones

	Coefficient	exp(β)	95% CI (LL, UL)		*P*-value	AIC	
Fitted model	*β* (1: lowest)	1.162	0.522	2.587	0.713	126.6	
	β (2: middle low)	0.683	0.431	1.085	0.106		Trend *P* =0.191
	β (3: middle high)	1.076	0.814	1.424	0.606		
	β (4: highest)	1.183	0.806	1.738	0.391		
Null model		-	-	-	-	122.6	

AIC, Akaike's Information Criterion; CI, confidence interval; LL, lower limit; UL, upper limit.

n=59

Null: the model without explanatory variables and coefficients.

eTable 2. Results of Poisson regression with estimated municipal groups based on the absorbed dose ranges with maximum estimates among different scenarios for evacuation zones

	Coefficient	exp(β)	95% CI (LL, UL)		*P*-value	AIC
Fitted model	β (1: lowest)	1.162	0.522	2.587	0.713	124.6
	β (2: middle low)	0.551	0.313	0.969	0.039	
	β (3: middle high)	1.097	0.824	1.460	0.526	
	β (4: highest)	1.165	0.832	1.630	0.374	
Null model		-	-	-	-	122.6

Trend *P* =0.127

AIC, Akaike's Information Criterion; CI, confidence interval; LL, lower limit; UL, upper limit.

n = 59

Null: the model without explanatory variables and coefficients.

eMaterial 3. Comparison of rate ratio estimations of incidence between Poisson regression and the complementary log-log binomial model

Suppose a different parametrisation of the Poisson model using a constant term, γ_0 as follows:

$$o_i \sim Poisson[r_i e_i],$$

$$\ln(r_i) = \gamma_0 + \sum_{k=2}^{4} \gamma_k z_{k,i},$$

where $\exp(\gamma_k)$ is considered as the estimate of rate ratio of the incidence of the *k*th group compared to the first group (lowest absorbed dose group). The result based on the reparametrized model is shown in eTable 3.

The regional absorbed dose variable can be used as an explanatory variable in the complementary log-log binomial regression, as shown in the eTable 4. It is worth noting that the rate ratio estimates obtained by the Poisson regression and the complementary log-log binomial regression agree to three significant digits.

eTable 3. Result of the Poisson regression with estimated municipal groups based on absorbed dose ranges (estimates of rate ratios)

Coefficient	exp (γ)	95% CI (LL, UL)		P-value
γ_0 (intercept)	1.162	0.522	2.587	0.713
γ_1 (1: lowest- Ref.)	1.000	-	-	-
γ_2 (2: middle low)	0.474	0.178	1.262	0.135
γ_3 (3: middle high)	0.907	0.388	2.120	0.821
γ_4 (4: highest)	1.067	0.448	2.540	0.884

AIC, Akaike's Information Criterion; CI, confidence interval; LL, lower limit; UL, upper limit.

n=59, AIC=123.6

Null: the model without explanatory variables and coefficients.

eTable 4. Results of the discrete survival model using complementary log-log binomial regression with the variable of estimated municipal groups based on absorbed dose ranges

	exp (Coef.)	95% CI (LL, UL)		P-value
α	2.20×10^{-10}	4.88×10^{-12}	9.91×10^{-9}	<0.001
β (round of TUE: second=0, third=1)	0.461	0.300	0.708	<0.001
β (Sex: male=0, female=1)	1.283	0.859	1.915	0.223
β (log(age in years))	23.971	9.909	57.985	<0.001
β (log(BMI in kg/m^2))	5.201	1.552	17.431	0.008
β (group 1: lowest- Ref.)	1.000	-	-	-
β (group 2: middle low)	0.474	0.178	1.263	0.135
β (group 3: middle high)	0.908	0.388	2.124	0.823
β (group 4: highest)	1.068	0.448	2.546	0.882

BMI, body mass index; CI, confidence interval; Coef., estimate of coefficient; LL, lower limit; UL, upper limit; TUE, thyroid ultrasound examination.

n=439,324, deviance=1693.1
